# Harnessing the Therapeutic Potential of Pomegranate Peel-Derived Bioactive Compounds in Pancreatic Cancer: A Computational Approach

**DOI:** 10.3390/ph18060896

**Published:** 2025-06-15

**Authors:** Rita Majhi, Sagar Kurmi, Hilal Tayara, Kil To Chong

**Affiliations:** 1 Department of Electronics and Information Engineering, Jeonbuk National University, Jeonju-si 54896, Jeollabuk-do, Republic of Korea; majhirita@jbnu.ac.kr (R.M.); kurmisagar@jbnu.ac.kr (S.K.); 2 School of International Engineering and Science, Jeonbuk National University, Jeonju-si 54896, Jeollabuk-do, Republic of Korea; 3 Advanced Electronics and Information Research Center, Jeonbuk National University, Jeonju-si 54896, Jeollabuk-do, Republic of Korea

**Keywords:** pomegranate peel, pancreatic cancer, network pharmacology, molecular docking, molecular dynamics simulation

## Abstract

**Background/Objectives**: Pomegranate *(Punica granatum)* peel, often discarded as waste, contains abundant bioactive compounds such as polyphenols, vitamins, flavonoids, tannins, anthocyanins, and many more. This contributes to remarkable bioactivities, including anticancer, anti-inflammatory, antioxidant, antibacterial, and antifungal properties. Pancreatic cancer is a deadly cancer with a 9% survival rate. Its aggressiveness, invasiveness, quick metastasis, and poor prognosis significantly decrease the survival rate. Thus, we aim to explore pomegranate peel as a possible alternative medication for treating pancreatic cancer through virtual methods. **Methods**: Firstly, bioactive compounds were collected from multiple databases and screened for oral bioavailability (OB) ≥ 0.3 and drug likeness (DL) ≥ 0.18 scores. Simultaneously, network pharmacology was employed to extract the most probable targets for pancreatic cancer. Further computational analyses were performed, including molecular docking, molecular dynamics simulation, and in silico pharmacokinetics evaluation. **Results**: Consequently, the top 10 key targets from network analysis were AKT1, IL6, TNF, SRC, STAT3, EGFR, BCL2, HSP90AA1, HIF1A, and PTGS2. However, only AKT1, EGFR, BCL2, HSP90AA1, and PTGS2 exhibited strong binding affinities with pomegranate compounds, which are significantly declared in affected cells to enhance cancer progression. Outcomes from molecular dynamics simulations, particularly RMSD, RMSF, hydrogen bonding, and radius of gyration (Rg), confirmed stable interactions between 1-O-Galloyl-beta-D-glucose, epicatechin, phloridzin, and epicatechin gallate with respective target proteins. **Conclusions**: This suggests that pomegranate peels hold anticancer bioactive compounds for treating pancreatic cancer. Surprisingly, most compounds adhere to Lipinski’s and Pfizer’s rules and display no toxicity. However, as this study relies entirely on computational methods, experimental validation is necessary to confirm these findings and assess real-world efficacy and potential side effects.

## 1. Introduction

Pancreatic cancer (PC) is a fatal malignancy where an uncontrolled mass of cells is found in pancreatic tissue. Globally, PC is regarded as seventh most prevalent type of cancer, with a larger percentage of cases occurring in developed countries [1]. Several factors contribute to PC in humans, including abnormal microbiological metabolism, blood glucose, aging, alcohol consumption, smoking, obesity, diabetes, hereditary factors, polluted air and water, and poor diet [2,3]. Due to the absence of specific symptoms, most patients are found to be diagnosed in the late stages of the disease, which is commonly known as late-stage PC. The survival rate of PC patients is less than 10 % compared to other types of cancers [4]. Certain treatment methods, such as surgery and chemotherapy, have shown limited success in improving prognosis. As per GLOBOCAN 2020, approximately 495,773 new pancreatic cancer cases were recorded in 2020, which is 2.6 percent of all new cancer cases in 2020, as reported by the Agency for Research on Cancer. In total, 466,003 deaths were reported, representing 4.7% of the total cancer-related mortality rate [5].

Natural plant-based compounds have become a viable substitute due to their distinct biological and pharmacological properties [6,7]. In recent years, people have been widely utilizing prior knowledge about bioactive products because of their anti-inflammatory, antibacterial, anticancer, radioprotective, anti-tumor properties and lesser toxicity [8]. It has also been found that natural products can cause apoptosis in PC cells as they regulate the expression of protein kinases, mitochondrial pathway, or genes that are involved in apoptosis. They can also inhibit the angiogenic process, limiting the growth and spread of cancer cells by obstructing the blood supply to the tumor cells [7].

Pomegranate (*Punica granatum*) is widely recognized for its health benefits, primarily attributed to higher concentrations of phytochemicals [9,10,11]. However, this research focused on pomegranate peels (PPs), which are considered to be non-edible parts of pomegranates (PG) and treated as waste but have potential sources of bioactive ingredients such as polyphenols, flavonoids, anthocyanins, organic acids, alkaloids, and hydrolyzable tannins [12,13]. Previous research has demonstrated that the peels of pomegranates possess anti-inflammatory, antimicrobial, anticancer, antioxidant, and cardioprotective properties [14,15,16]. Moreover, it has nutraceutical properties contained in minerals, fats, carbohydrates, proteins, and fibers [8]. There is evidence that pomegranate peel polyphenols, flavonoids, and anthocyanin impact several diseases, including diabetes and metabolic syndrome, cardiovascular diseases, male infertility, Alzheimer’s disease, gastrointestinal diseases, and cancer inflammation [17]. Hence, this study involves network pharmacology to find the most relevant pancreatic cancer target protein for pomegranate-derived compounds and to investigate drug interactions at different target sites and related diseases by constructing “drug-gene target disease” and its associated pathways. Additionally, pharmacokinetic properties and toxicity assessments of all compounds are carried out. Molecular docking is employed to predict the binding pose between two molecules, a ligand and receptor. Further, we determine how well molecules fit into the receptor pocket by calculating the binding affinity, which is expressed as docking score along with key interacting amino acids of proteins with compounds. Simultaneously, molecular dynamic simulation is used to verify the stability of protein and compound binding affinity.

## 2. Results

### 2.1. Finding Active Compounds and Common Target Proteins

We collected 143 compounds from pomegranates through various databases and literature reviews. The final compounds obtained were 75 after screening from oral bioavailability (OB) and drug likeness (DL), presented in Table S1. This is crucial for early drug design because it provides information about the effectiveness of the drug in various body systems and physicochemical properties, whether it reaches the target site or not [18]. Furthermore, toxicity was analyzed, and the final compounds were 15, as shown in Table 1. Swiss target prediction and STICH databases resulted in 698 target genes of compounds. Similarly, from the OMIM and gene cards databases, we gained 233 and 13,780 target genes related to pancreatic cancer, respectively. Then, duplicate genes in OMIM, gene cards, and compound targets were removed. Venn analysis of compound targets and PC targets was then carried out.

**Figure 1:**
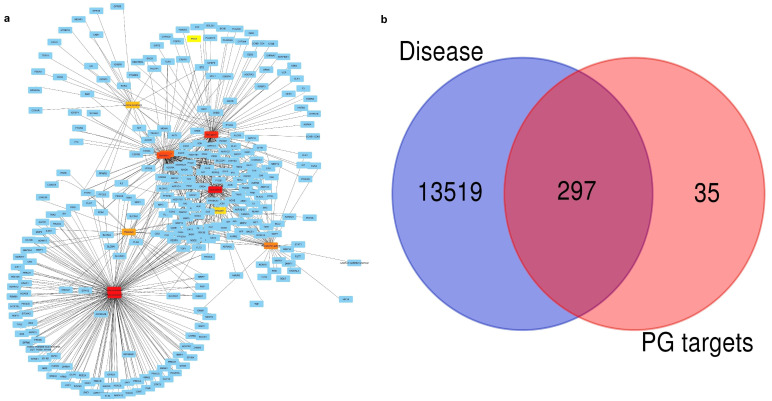
Pomegranate–Pancreatic Cancer. (**a**) Top 10 compound targets highlighted in yellow, red, orange and target proteins are highlighted in blue colors; (**b**) Intersection of target genes between disease and pomegranate.

As a result, Figure 1a illustrates the interactions of predicted protein targets (blue nodes) and the PG compounds identified as shades of red to orange, indicating that each edge represents an interaction, which suggests that the compound may have multiple biological targets that it may act upon. Also, the different degrees of connectivity between the central hubs are differentiated (yellow to dark red): darker nodes represents greater interaction with target proteins. Therefore, PG compounds exhibit multi-target properties, which are important characteristics of complex disease treatment approaches the top ten compound targets outlined in Figure 1a. Similarly, Venn analysis was developed to compare the overlap between disease-related genes and PG target genes. Out of the 13,519 disease-associated genes, 297 were found to have cross-relationships with the 332 PG target genes, which indicates a significant link between PG compounds and disease-relevant genes. Due to their role in disease processes, the intersecting 297 genes share a great deal of therapeutic potential. These overlapping genes could be used to identify potentially drug candidate and disease-associated targets, which could guide future studies for the validation as depicted in Figure 1b.

### 2.2. Investigation of Protein–Protein Network Interactions

**Figure 2:**
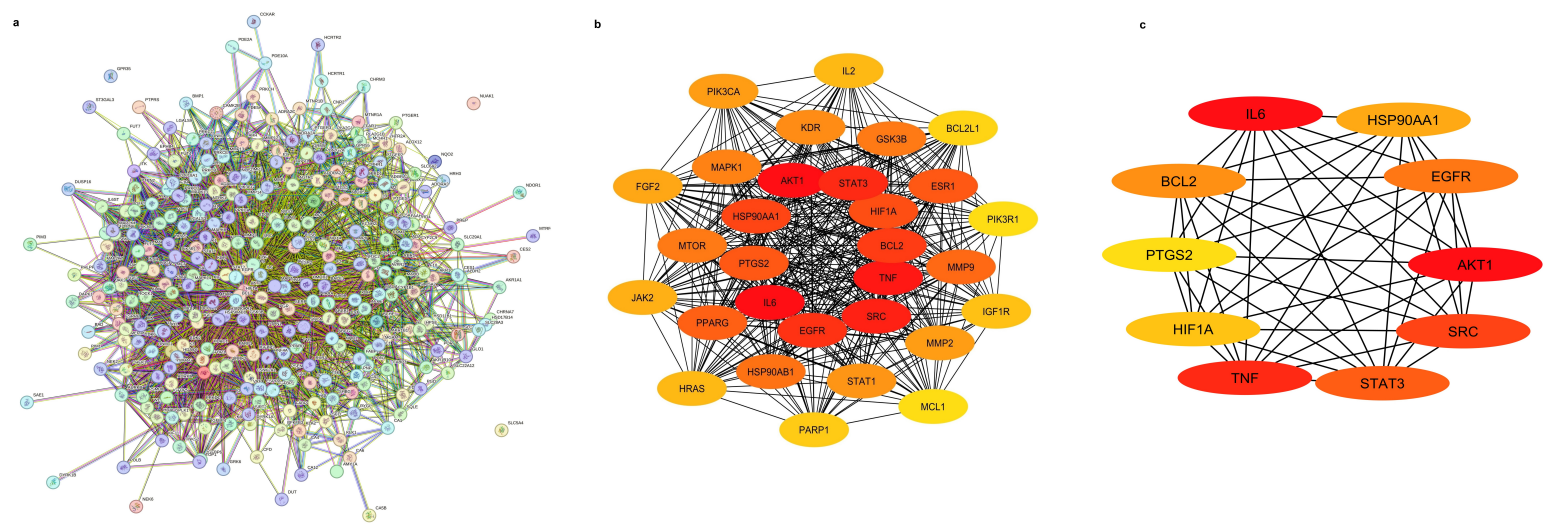
Protein–protein interaction network. (**a**) Interaction nodes and edges; (**b**) Top 30 gene interactions obtained from (**a**); (**c**) Top 10 genes responsible for PC management.

The 297 common genes from the Venn analysis were imported into STRING web server. These common genes were from pomegranate compound targets and pancreatic cancer targets. Our data demonstrated 297 nodes and 4415 interaction edges as shown in Figure 2a. The network was linked with Cytoscape software 3.10.2 for topological analysis and to obtain the top key genes responsible for pancreatic cancer treatment. The significance of each node in protein–protein interaction was characterized using parameters of degree, closeness centrality (CC), and betweenness centrality (BC) and degree of connection presented in Table 2. The top 30 and core target proteins shorted from 30 targets are demonstrated in Figure 2b and Figure 2c, respectively.

### 2.3. Go and KEGG Terms Analysis

**Figure 3:**
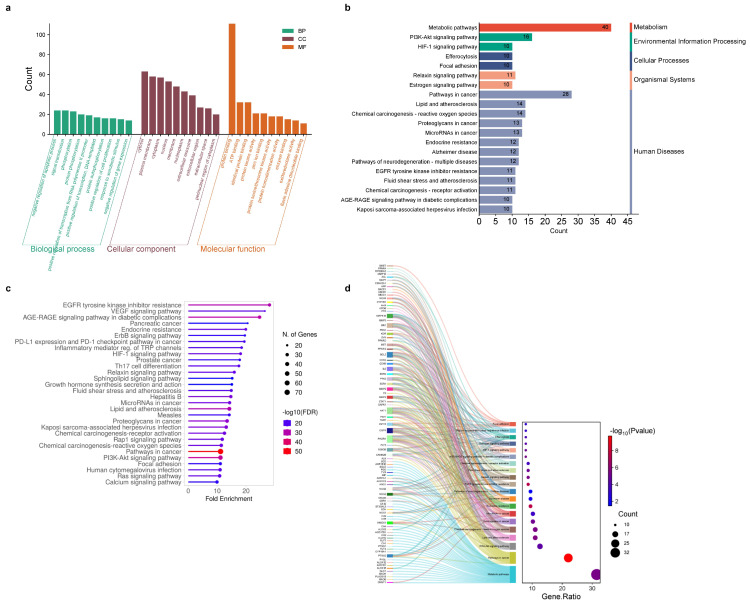
GO and KEGG analysis. (**a**) Top 10 Go enrichment terms; (**b**) The histogram diagram of genes in each pathway; (**c**) Top 30 KEGG pathways involved in PC; (**d**) Sanky and Bubble diagram of KEGG pathways for hub targets.

**Figure 4:**
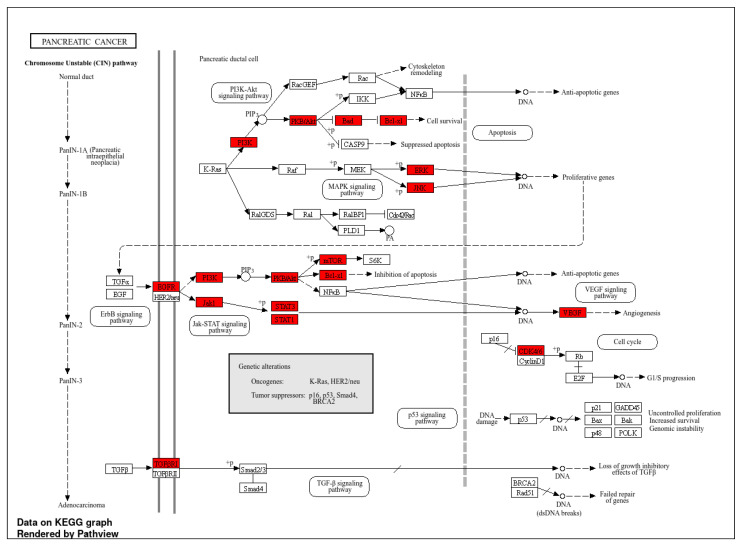
Pancreatic cancer pathways and key proteins highlighted in red color.

Go and KEGG analyses aim to collect biological information and important signaling pathways that control biological processes. Go enrichment analysis is illustrated in Figure 3a. In biological process (BP), most genes are involved in decreasing of apoptotic process and signal transduction. Assembled genes were present in cytosol, plasma membrane, cytoplasm, and nucleus. Figure 3b illustrates the higher number of genes participating in metabolic pathways and pathways in cancer, which accounted for 40 and 28 gene numbers, respectively. Lastly, the genes involved in molecular function were protein binding, ATP binding, identical protein binding, protein kinase activity, and zinc ion binding. Furthermore, the top 30 signaling pathways shown in Figure 3c participate in pancreatic cancer and the P13/Akt signaling pathaway. The relations diagram between the top 20 pathways and the number of significant gene counts are mostly related to metabolic pathways, pathway in cancer, and P13/Akt pathways, as shown in Figure 3d. The key pathways and genes involved in pancreatic cancer are depicted in Figure 4, which offers information about crucial target proteins for treating pancreatic cancer.

### 2.4. Docking Score of Core Targets with Compounds

**Figure 5:**
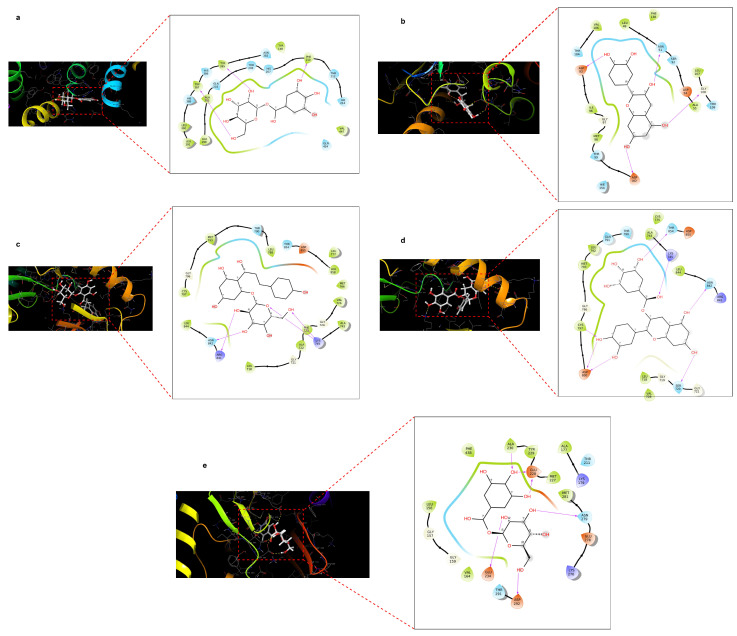
Molecular interactions between protein and ligands. (**a**) 1-O-Galloyl-beta-D-glucose compound interaction with PTGS2 protein (PDB ID: 5F19); (**b**) Epicatechin compound interaction with HSP90AA1 protein (PDB ID: 6TN5); (**c**) Phloridzin compounds interaction with EGFR protein (PDB ID: 8A27); (**d**) Epicatechin gallate interaction with EGFR protein (PDB ID:8A27); (**e**) 1-O-Galloyl-beta-D-glucose compound interaction with AKT1 protein (PDB ID:4GV1).

Molecular docking of selected pomegranate compounds was performed with the help of Schrodinger platform. It provides information about binding affinity and specific interactions between proteins and compounds. Among 15 compounds of pomegranate peels, only 11 compounds showed docking score of less than (<−6 kcal/mol). We took a score of less than or equal to −6 kcal/mol as initial filtering criteria to prioritize a manageable number of ligands for further analysis [19]. Moreover, a docking score of less than −8 kcal/mol was obtained in catechin, genistein, epicatechin gallate, and astragalin complex with HSP90AA1, EGFR, and protein PTGS2. However, other compounds did not show any good binding affinity scores. The docking scores of all compounds are shown in Table 3. However, proteins such as IL6, TNF, SRC, STAT3, and HIF1A could not bind with any selected compounds of pomegranate peels, and compounds that showed promising interactions with proteins are mentioned in Figure 5. In Figure 5a, the ligand forms multiple H-bonds with residues TRP387, TYR385, and PHE210, while the surrounding residues provide hydrophobic support and a polar environment that may enhance specificity. These types of interactions are a hint of deep and specific binding within the binding pocket which is likely an active region of the receptor. Also, as depicted in Figure 5b, the ligand forms four key hydrogen bonds with negatively charged residues (ASP93, ASP102), polar residues ASN51, and water-bridge with residues GLY108, reinforcing strong and stable binding to the ligand. The surrounding hydrophobic bond highlighted in green and polar residues (light blue) help in maintaining an optimal binding environment, potentially contributing to selectivity and binding stability. The ligand is connected to polar and charged residues like ASN842, ARG841, and LYS745 by multiple hydrogen bonds, as shown in Figure 5c. Hydroxyl groups are primarily responsible for these interactions. The ligand aliphatic side chains are surrounded by a potent hydrophobic environment, which improves nonpolar stabilization. The diverse set of interactions suggests a highly complementary binding pocket, enhancing both polar and nonpolar contacts for high binding affinity. Moreover, in Figure 5d, the ligand is docked with several hydrogen bonds, especially with charged and polar residues (ASP800, ASN842, LYS745, and SER720). Hydrophobic residues provide additional stabilization via nonpolar interactions. The ligand is well positioned within a pocket formed by a mix of polar and hydrophobic environments, resulting in enhanced binding affinity and specificity. Finally, Figure 5e shows that the ligand binds to residues ALA230, GLU228, ASN279, GLU234, and ASP292 to form five strong hydrogen bonds. The ligand is stabilized in the binding pocket by the hydrophobic residues TYR229 and MET227. The binding pocket offers a well-balanced mix of polar and nonpolar environments which enhances the binding affinity, specificity, and also overall stability of protein–ligand complexes which is crucial for molecular identification and therapeutic targeting. Among aforementioned interactions, the AKT1-1-O-galloyl-beta-D-glucose complex displays extensive interaction compared to other interactions.

### 2.5. Molecular Dynamic Simulation

**Figure 6:**
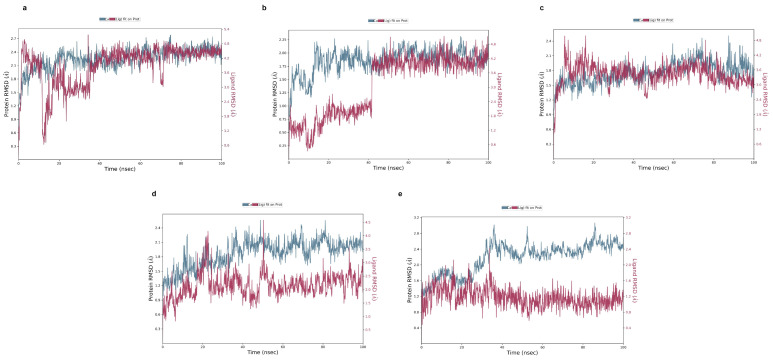
RMSD of all compounds. (**a**) 1-O-Galloyl-beta-D-glucose with PTGS2 protein; (**b**) Epicatechin with HSP90AA1 protein; (**c**) Phloridzin with EGFR protein; (**d**) Epicatechin gallate with EGFR protein; (**e**) 1-O-Galloyl-beta-D-glucose with AKT1 protein.

Molecular dynamics simulation confirms the stability between compounds and proteins by evaluating RMSD, protein–ligand contact, hydrogen bonding, RMSF, and radius of gyration. We performed molecular dynamics simulations of all top-ranked docking scores less than or equal to −6.00 kcal/mol as a threshold; they are given in Table 3. The lower the RMSD value, the higher the stability between complexes. Among many, some protein–ligand complexes reveal promising outcomes, which is explained in a section below. Firstly, in Figure 6a, the PTGS2-1-O-Galloyl-beta-D-glucose complex exhibited initial fluctuations between 1.5–2.3 Å over 15ns. Afterwards, protein RMSD graph stabilized gradually and levitated between 2.0 and 2.7 Å until the end of the simulation. So, an RMSD of 2.7 Å means the protein, especially the backbone of the protein, has shifted to 2.7 Å from its initial position throughout the time of simulation, which means the binding ligand did not undergo extreme conformation changes [20,21]. The value obtained from RMSD tells us about the nature and stability of the protein–ligand complex. In the initial time, when the ligand came in contact with the protein, it underwent some adjustment and finally bound to the pocket of protein either strongly or weakly. Therefore, it suggests that there were minor fluctuations but an overall stable trend after 30 ns. However, the ligand RMSD started with high fluctuations, dropped as low to 0.6 Å, and spiked over 5 Å before 40 ns. After that, the ligand stabilized within the 4.2–4.8 Å range, illustrating moderate mobility with in the pocket with some conformational shifts. Secondly, the complex in Figure 6b was initially low around 1.6 Å, and it rose and stabilized near 2 Å at around 20 ns. There was minor fluctuation observed but the graph maintained equilibrium after 30 ns suggesting the stability of protein throughout the simulation. However, looking at the ligand graph, there was a sharp rise from 0.6 Å to 3.6 Å in the first 20 ns. Afterwards, the graph maintained stability around 3.6–4.2 Å. Similarly, the complex of EGFR- phloridziz fluctuated between 1.2 and 2.4 Å. The protein was observed to be fluctuated throughout time. However, the fluctuation was minor with stable RMSD average range from 1.6 to 2.2 Å. The ligand maintained a stable RMSD graph, within 2.0–3.6 Å most of the time, as shown in Figure 6c. Moreover, in Figure 6d, the RMSD graph reached its highest fluctuation up to 2.4 Å. However, the graph initially elevated from approximately 1.2 Å to 2.2 Å. Afterwards, the protein reached its stability around 2.0–2.3 Å after 25 ns with minor fluctuations. Therefore, the protein structure reached equilibrium and remained steady thereafter. Looking at the ligand graph, from 40 ns, the graph settled in the 2.0–3.0 Å range. The interaction fraction exceeded 1.6. Lastly, in the complex in Figure 6e, the RMSD graph displays a steady increase from 1.8 Å to 2.6 Å by 40 ns. After 40 ns, the graph stabilized between 2.4 and 2.8 Å with minor fluctuation which is an acceptable range. Moreover, the ligand RMSD graph remained stable throughout between 1.0 and 1.4 Å, suggesting strong binding affinity. These indicate that pomegranate compounds form strong, stable interactions with target pancreatic cancer proteins. The results from post-molecular-dynamics simulations showed that reorientation of the ligand occurred during the simulations, forming new interactions like water-mediated hydrogen bonds and additional residual contacts compared to original docking poses. This might be due to the movement and adjustments of the ligand in protein residues in physiological simulation conditions (Figure S7).

**Figure 7:**
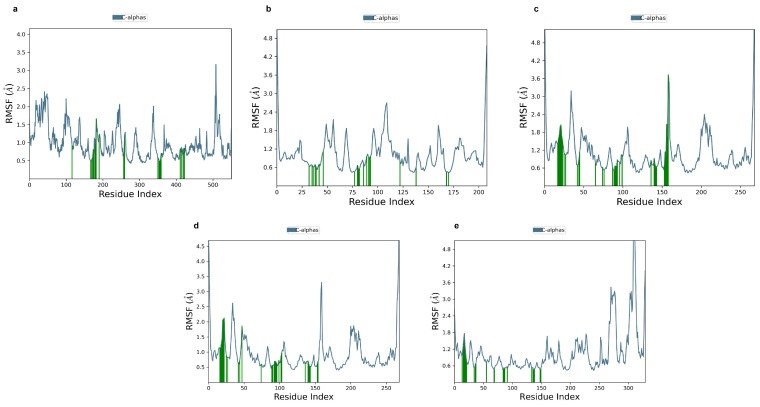
RMSF (**a**–**e**) illustrates the protein–ligand interaction, highlighting (green lines) the key residues that are involved in the stabilizing ligand within the binding pocket of the protein. (**a**) 1-O-Galloyl-beta-D-glucose with PTGS2 protein; (**b**) Epicatechin with HSP90AA1 protein; (**c**) Phloridzin with EGFR protein; (**d**) Epicatechin gallate with EGFR protein; (**e**) 1-O-Galloyl-beta-D-glucose with AKT1 protein.

**Figure 8:**
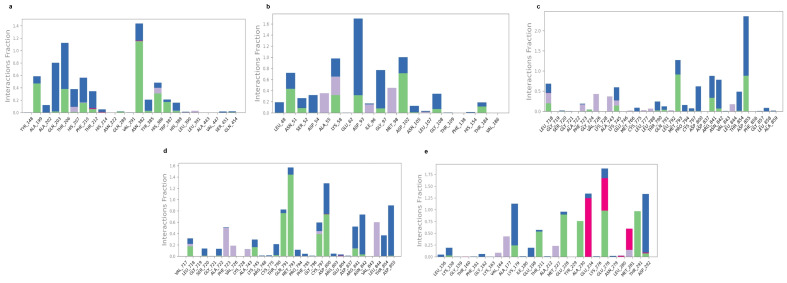
Protein–ligand contact: Green (H-bonds); Grey (Hydrophobic); Pink (Ionic); Blue (Water bridge); (**a**) 1-O-Galloyl-beta-D-glucose with PTGS2 protein; (**b**) Epicatechin with HSP90AA1 protein; (**c**) Phloridzin with EGFR protein; (**d**) Epicatechin gallate with EGFR protein; (**e**) 1-O-Galloyl-beta-D-glucose with AKT1 protein.

The second criterion for stability observation is an RMSF graph, which offers information of individual amino acid flexibility throughout simulation. Our data revealed that both stable and flexible segments in are present in the structure. The majority of fluctuations occurred in the terminal regions, where the RMSF values were frequently in the range of 3.5 to 4.8, indicating extremely high mobility in these regions. On the other hand, the structural parts of the proteins, indicated by the green bars, displayed lower RMSF values (around 0.5 to 1.5 Å), reflecting their structural stability. As seen in Figure 7e, the core was more stable, especially between residues 50 and 200, whereas the values in Figure 7a,d are more flexible. On the other hand, in Figure 7c, a change in motion appears around residue 150 as opposed to Figure 7b, where the data are relatively rigid except at the ends. Therefore, the overall structure of the protein remains intact, with the majority of flexibility concentrated in the terminal and loop regions of the protein. Figure 8 illustrates the different bonding types between ligand and receptor along with the frequency of interactions during simulation. Initially, the binding in Figure 8a was facilitated by THR 206, Thr 210, and ASN 382 that formed hydrogen bond frequently and maintained interaction fractions above 1.0, indicated in green and blue colors, respectively. Further, GLN 203, ALA 199, PHE 213 presented mixed bonding. In addition, in Figure 8b, several residues (LYS58, ASP93, and ASP102) exceed the 1.0 interaction fraction and have multiple interactions; ASP 93 mostly made a hydrogen bond and some hydrophobic angle. Other significant contributers, such as ASN 51, LYS58, GLY 97, and ASPO 102, have an interaction fraction around 0.6. In addition, residue ASP855, which has an interaction fraction over 2.0, was highly responsible for stability maintenance between EGFR and phloridzin as shown in Figure 8c. Other residues involved in binding include MET793, ARG 841, and ASN 842. Residues like MET 793 and ASP 100 reach interaction fraction above 1 in Figure 8d. They are the most prominanat binders, forming hydrogen along with hydrobhobic contacts. Meanwhile, diverse interaction types including ionic and hydrophobic with multiple residues and frequency of interaction above 1.5 are illustrated in Figure 8e. GLU 278,GLU 234, and ASP 292 have higher interaction fractions and involve ionic bridges. Further, THR 291 retained stability by hydrogen bond generation. So, all these key residues play a crucial role in binding 1-O-Galloyl-beta-D-glucose with AKT1.

Additionally, protein structure analysis data reflect the stability and behavior of protein during simulation. Notably, graphs (b–d) of Figure S1 have higher proportion of secondary structure elements (SSE) around 47–50%, signifying the maintenance of the native secondary structure, whereas graphs (a,d), Figure S1, have SSE significantly below 41%, suggesting flexibility and partial unfolding. Moreover, the distributions of helix and strand are uniform and stable in the simulation. In a similar way, the radius of gyration (Rg) illustrates a minor orientation shift but no major folding or collapse and structural stability; see Figure S1, right side, green-color graph.

### 2.6. Ligand-Free Protein RMSF

From root square fluctuation (RMSF) of apo (unbound) protein data, the apo protein was more flexible than the ligand-bound protein especially in loop regions and the N terminal. For instance, ligands like 1-O-Galloyl-beta-D-glucose and epicatechin reduced the variation in binding residues, meaning it improved the structural stability of binding proteins. Due to ligand interactions, some regions showed increased flexibility, while others displayed more rigidity, as represented in Figure S6. This suggests that interacting ligands cause dual effects in proteins. These results demonstrate that ligand binding not only stabilizes the specific binding sites but also induces long-range dynamic changes across the protein structure, highlighting the allosteric nature of interactions.

### 2.7. In Silico ADME, Toxicity, and Lipinski and Pfizer’s Rule

ADME analysis offers valuable information on compounds in the early stage of drug development. Table 4 demonstrates the pharmacokinetic characteristics of compounds. Our result illustrates that all compounds are water soluble, ranging from 0.045 to −7.641. Additionally, the volume distribution of drugs was found to be between 0.04 and 20 L/kg, indicating that our compounds have greater distribution in tissue rather than plasma. Interestingly, toxicity results show that all compounds are free from hepatotoxicity, immunotoxicity, mutagenicity, and cytotoxicity. Lastly, we observed Lipinski’s and Pfizer’s rules to ensure whether our molecules hold drug-like properties concerning physicochemical properties. Table 5 shows Lipinski and Pfizer’s rules and the toxicity of the selected compounds.

## 3. Discussion

Pomegranate is recognized as a fruit with excellent health benefits due to its bioactive properties. There is no doubt that pomegranate fruit has many advantages, but the peel also provides a wide variety of benefits, just like the fruit [22]. Thus, this fascinating attribute could contribute to the treatment of certain diseases like pancreatic cancer (PC), a highly aggressive malignancy with limited therapeutic options [13,23]. The treatment of PC remains challenging due to extreme aggressiveness, poor prognosis, and lack of early-stage symptoms. A network-based approach was performed to identify bioactive compounds from pomegranate peels that could serve as potential therapeutic agents for PC. Before choosing the final PG compounds, we screened the compounds DL and BO. This is an important step in determining the properties of our compounds of interest with positive and negative sets of drugs and whether our products will reach the site of drug action [24]. Our analysis identified 297 common targets shared between pomegranate-derived compounds and PC-related proteins (Figure 1b). This common target is useful for finding the best target proteins for further analysis. Protein–protein interaction data revealed the top 10 key target proteins (Table 2), indicating these targets might be responsible for pancreatic cancer. However, the docking reports demonstrated that five proteins (AKT1, EGFR, BCL2, HSP90AA1, PTGS2) exhibited strong binding with pomegranate compounds with good scores. Previous studies have explored the effects of catechin and its derivatives on disease-relevant targets including BCL2 and HIF1A as a key target. This research mentioned molecular docking of catechins with receptors similar to those we collected [25]. Similarly, Rehan et al. [26] investigated cancer therapeutic properties of catechin and epicatechin with epidermaal growth factor receptor (EGFR) involved in cancer progression. This study sketched that pomegranate peel compounds like 1-O-galloyl-beta-D-glucose, Phloridzin, epicatechin gallate, and epicatechin show promising interaction with AKT1, EGFR, HSP90AA2, and PTGS2, as confirmed by RMSD, RMSF, and interaction fractions (Figure 6, Figure 7 and Figure 8). For other compounds like quinic acid and kaempferol, interactions are given in Figure S2. Most compounds possess binding energy ≤−6.0 kcal/mol [27] while the reference drugs tanespimycin, erlotinib, afuresertib, and celecoxib docking score ranges from −5.48 to 8.07 kcal/mol with proteins HSP90AA1, EGFR, AKT1, and PTGS2, respectively, as shown in Table S2. The RMSD graph of the selected complex indicates less fluctuation or movements of proteins and ligands during contact, and deviation is in acceptable range from 2.25 to 3.2 Å compared to the reference drug; Figure S3. When the protein structure is dramatically changed, the protein loses its native structure (secondary and tertiary structure), indicating possible denaturation and incomplete equilibration. Loss of stability can also be confirmed by higher fluctuation and flexibility in protein structure, losing stable hydrogen bond, which is not good for complex stability maintenance. So, our protein conformational changes are not drastic, and stability is maintained. Comparing the RMSD value with that of reference drugs, the range is between 2.0 to 3.5 Å, occasionally reaching 4 Å, which reflects noticeable confrontational shifts. In contrast, our results displayed lower and more consistent values, signifying enhance structural stability. Further, the RMSF result of reference drugs indicated that some binding residues have higher fluctuation compared to test PG compounds, and protein–ligand contact revealed that while both the control and test compounds displayed various interactions, the pomegranate ligands demonstrated higher interaction fraction, often reaching a value of one. These data suggest more stable anchoring in binding residues. A significant number of hydrogen bonds, hydrophobic bonds, and water bridges can be observed in the interaction diagram (Figure 5 and Figure 8). Interestingly, epicatechin, 1-O-galloyl-beta-D-glucose, epicatechin gallate, and pholoridzin can bind with this carcinogenic protein, which might play a pivotal role in PC suppression (Figure 6 and Figure S1). The EGFR-epicatechin gallate and 1-O-galloyl-beta-D-glucose-AKT1 complex have widespread interaction with cancer-associated targets, while others have fewer interactions. This bonding is crucial for effective stability and good affinity in drug discovery.

Additionally, our GO and KEGG analyses highlight several pathways and the involvement of genes in various functions. The high frequency of genes in the biological process mainly participate in apoptotic regulation process and cellular signaling along with significant molecular functions like binding and enzymatic activity. The major process primarily takes place in the cytosol. The highest number of genes were associated with metabolic and cancer-related pathways like P13/Akt, which is known for cell growth regulation, apoptosis, survival, metabolism, and proliferation [28,29,30]. The top enriched pathways include AGE-RAGE, epidermal growth factor (EGFR), and VEGF, which are highly expressed in pancreatic cancer. The outcomes from our analysis indicate their importance in tumor growth and drug resistance because these pathways are linked to cancer progression and angiogenesis (Figure 3). So, targeting these pathways ultimately initiates apoptosis and reduces proliferation, and this can be achieved using pomegranate compounds, which could be a good therapeutic candidate in cancer treatment. Oncogenic genes like *EGFR* and *AKT1* are responsible for metastasis, cell proliferation, and blood vessel formation and are highly expressed in pancreatic cells, leading to poor prognosis [31]. The triggering of *EGFR* causes the activation of myofibroblasts, leads to the secretion of various factors, and causes the progression of cancer cells [32]. Similarly, *HSP90AA1* is known to enhance tumor aggressiveness, while *PTGS2* (encoding COX-2) promotes apoptosis resistance, proliferation, inflammation, and metastasis of cancer cells [33]. Additionally, some investigations into the role of flavonoids in modulating cancer signaling cascade also exemplifies that this natural compounds plays a significant role in disrupting the cancer signaling cascades, causes inflammation, and is involved in the apoptosis process to destroy the cancer cells [34,35]. This can be achieved by suppressing vital pathways like P13/Ak and EFGR, whose main role is enhancement of cancer development. Further, research findings suggest that pomegranate compounds can inhibit or suppress this carcinogenic protein and halt cancer cell progression.

Finally, to assess the drug-like character of compounds, ADMET (Adsorption, Distribution, Metabolism, Excretion, and Toxicity) analysis was conducted. Remarkably, most of the compounds of pomegranate are free from toxicity. This property make compounds more valuable for the drug development process. Besides this, evaluation of compounds via Lipinski’s and Pfizer’s rules was achieved; 15 compounds met these criteria Table 5. However, epicatechin gallate and kaempferol displayed moderate solubility but high tissue distribution (−4–0.5 log mol/L) [36]. The rest of the compounds had good water solubility. For optimal absorption, the drug should be dissolved sufficiently in the blood circulation system; otherwise, it affects the drug discovery process [37]. All compounds exhibited no BBB permeability and higher human intestinal absorption ≥ 30% (HIA), indicating higher bioavailability [38]. Certain selected compounds have a high clearance rate, which means they can be eliminated from the body quickly. Conversely, 1-o-galloyl-beta showed limited clearance, indicating prolonged retention within the body. As a result, a lower dosage is required to achieve therapeutic efficacy. Most compounds displayed poor intestinal permeability (low CaCO_2_) value. Additionally, our promising compounds, epicatechin, phlorizin, and 1-O-galloyl-beta-D-glucose, acted as anti-inhibitors of multiple CYP enzymes, except epicatechin gallate and kaempferol. All these satisfactory data from our research indicate that each compound of pomegranate peels carries highly valuable drug-like properties, signifying strength in the drug development process as well as for treatments of various diseases, including highly aggressive pancreatic cancer, by inducing apoptosis or autophagy and participating in cancer-related pathways. Additionally, natural-based drugs mitigate the health side effects compared to synthetic drugs that are available on the market. Moreover, people treat pomegranate peels as waste; consequently, this research minimizes the negative impact on the environment and concurrently adds the valorization of pomegranate peels.

Despite the promising computational outcomes, it is critical to consider the limitation of computational methodologies. Furthermore, the verification of computational outcomes can be achieved through in vivo and in vitro studies. Thus, based on our data, pomegranate compounds may interact with several pathways to play a significant role in PC inhibition. Because of the promising benefits of PG in various cancer inhibitions, the above compounds may be suitable for PC.

## 4. Materials and Methods

### 4.1. Screening of Bioactive Compounds

Bioactive compounds from pomegranate were extracted from different databases like Traditional Chinese Medicine Systems Pharmacology (TCMSP) https://www.tcmsp-e.com/tcmsp.php (accessed on 12 September 2024), Indian Medicinal Plants, Phytochemistry, Therapeutics https://cb.imsc.res.in/imppat/ (accessed on 12 September 2024), and literature review. The data obtained were filtered using oral bioavailability score (OB) ≥ 0.3 and drug likeness (DL) ≥ 0.18 thresholds [18]. Furthermore, using PubChem-ID, we recorded each compound’s detailed information.

### 4.2. Identification of Pancreatic Cancer and the Compound’s Target Genes

The compound target protein was collected by inserting canonical SMILE into Swiss Target Prediction http://www.swissadme.ch (accessed on 19 September 2024) and STITCH data http://stitch.embl.de/ (accessed on 25 September 2024). These two databases present each compound’s target proteins. Furthermore, the compound target probability was filtered with a value of 0.1. Similarly, disease target proteins were gathered from two online databases, Online Mendelian Inheritance in Man (OMIM) and Gene Cards. These databases have a collection of all genes related to disesaes. They were used to identify genes associated with pancreatic cancer. All the related target excel files were downloaded for further analysis.

### 4.3. Exploration of Overlapping Genes

Firstly, data retrieved from OMIM, gene cards, and pomegranate targets were combined and inserted into the Venn analysis website, https://bioinformatics.psb.ugent.be/webtools/Venn/ (accessed on 2 October 2024). The website provides information on common genes between OMIM, gene cards, and compound target genes.

### 4.4. Hub Gene Analysis Through Protein–Protein Network

The overlapping genes identified from the Venn analysis were analyzed using STRING 12.0 to see every interaction of each gene with high confidence scores. STRING is a biological database that is used to image protein–protein interactions and to obtain key proteins with biological pathways. To achieve this, selection parameters were set as multiple proteins and organism homo sapiens, and interactions were visualized through Cytoscape 3.10.2 which delivers the topological properties including nodes and relationship within the networks such as degree and betweenness closeness. The plugin Cytohubba identified the top 10 genes, and Centiscape calculated their network parameters.

### 4.5. Bioinformatics Technique for GO and KEGG Analysis

Observation of biological phenomenon on molecular-level function can be accomplished using Gene Ontology (GO) that delivers all the related reports on the role of protein. This was achieved by applying the DAVID database https://david.ncifcrf.gov/tools.jsp (accessed on 10 October 2024), which is the bioinformatics tool that interprets biological and functional annotation of the protein list. In the same way, important pathways involved during pancreatic cancer treatments were also found through DAVID, and results were looked at in SRplot. Screening condition *p* < 0.05 was used to obtain the main pathways and roles of the collected genes.

### 4.6. Molecular Docking of Hub Genes

Molecular docking helps to anticipate the most probable binding pose of a ligand with in the associated target receptor, and this can be achieved using Schrodinger (Schrödinger, 2024). The 3D structures of each compound and related proteins were downloaded from PubChem and RSCB-PDB databases, respectively. Regarding protein selection, we gathered high-resolution crystallographic structure without mutation. The higher resolution provided the co-ordinates of each atom in detail and aided in generating trustworthy binding affinity. The PDB IDs used were 4GV1 (AKT1), 1ALU (IL6), 2AZ5 (TNF), 1043 (SRC), 6NJS (STAT3), 8A27 (EGFR), 4MAN (BCL2), 6TN5 (HSP9OOA1), 7LVS (HIF1A), and 5F19 (PTGS2). Firstly, in PyMOL and PUResNet, a ligand binding pocket was chosen. The binding residues were selected by creating a 4 Å distance from a co-crystallized ligand in PyMol [39].

#### 4.6.1. Ligand Preparation

Using the LigPrep module, the imported structures were refined geometrically into Maestro, creating a single low energy. Consequently, the primary states of ionization and chiralities were determined at this stage. The maximum atom size and possible ionization state were set to 500 and Epik at pH 7.0 ± 2, respectively. Tautomer and steroisomer generation was allowed to generate different forms of ligand by position and different 3D arrangement of atoms. The confirmation of ligand structure was minimized using the OPLS4 force field. This provides improved parameterization for both proteins and small molecules, enhancing protein–ligand interactions and conformational flexibility for charged and polar groups. It also alleviates steric hindrance and achieves an ideal geometry by adjusting bond lengths, angles, and torsions, thereby indicating a more accurate receptor conformation. Also, this helps in energy minimization before the commencement of docking simulations [40,41].

#### 4.6.2. Protein Preparation and Docking Setup

At last, the imported protein was prepared in Maestro using the default option provided by the protein preparation wizard in Schrodinger, which includes the addition of missing hydrogen atoms, missing chain filling, assignment of bond order, protonation state adjustment using PROPKA at pH7.4, and Epik to predict protonation states and tautamers of molecules. The protein energy was suppressed with an OLSP4 force field. Furthermore, the grid dimension was generated at the centroid of selected residues. And inner and an outer box were set to 10 Å and 20 Å from the ligand center. This allowed the entire ligand to fit inside the grid [42]. This was achieved using the receptor grid generation module. Following grid formation, the standard precision mode (SP) docking was used with van der Waals scaling factor 0.80 and partial charge cutoff 0.15 for non-polar atoms. Moreover, nitrogen inversion, ring flexibility, torsion bias for functional groups, and the Epik tool were applied during docking. Numerous binding poses for individual ligands were maintained by setting a 30 kcal/mol window energy. Lastly, 10 poses per ligand were created to collect top glide score.

### 4.7. Validation of Molecular Docking

Using the Pymol visualization tool, the co-crystallized ligand attached in the active sites of proteins was separated and redocked into the same active sites of that protein. This was performed to validate the docking score and for reliability of the results.

### 4.8. Assessment of Protein–Ligand Complex Stability

Molecular dynamics (MD) simulation was performed using Desmond through the Schrodinger Maestro interface. Simulation offers information on how much the ligands and proteins are bound with each other and their stability, along with various inter-molecular interactions. Firstly, the top ranked docking score was taken for MD simulation, and during this, solvent MD package, a fixed OPLS4 force field, and simulation period at 100 ns were set. The protein–ligand complex was maintained with an orthorhombic periodic box. The buffer distance was set to 10 × 10 × 10 Å, and water was added to molecules using the TIP3P model. The sufficient charges like 0.15 M NaCl, Na^+^, and Cl^−^ were added to balance the complex systems. A constant number of particles as well as the pressure and temperature (NPT) ensemble method was used at 300.0 K temperature and 1.013 bar pressure, together with the relax model system before simulation [43,44]. The relax model was incorporated with a thermostat (Nose-hoover Chain with relaxation time 1.0 ps) and a barostat method (Martyna–Tobias Klein with relaxation time 2.0 ps). In addition, we applied an RESPA multiple time step integrator with a 2 fs time for bonded, 2 fs for near, and 6 fs for far steps, and a 9.0 Å cutoff was used as a short-range integrator. At last, a trajectory interval of 4.8 ns, 10 ps of energy, and a solvent box of a 10 Å size were set up [45]. Afterward, a simulation was run usingthe Desmond module of Schrodinger, and the stability of complexes was assessed using graphical parameters obtained from simulation results.

### 4.9. In Silico Pharmacokinetics (ADME) and Toxicity Assessment

ADMET is a crucial property required before developing therapeutic drugs. Drugs must have a good pharmacokinetic profile to be effective for diseases. For pharmacokinetic analysis, we employed ADMET Lab 3.0. [38,46]. It screened the compound properties like absorption, distribution, metabolism, excretion, and toxicity in the body after consumption and also offered ideas for the early drug discovery process. The ProTox 3.0 server screened information about the toxicity of selected pomegranate compounds. Furthermore, we checked whether compounds followed Lipinski’s and Pfizer rule for drug-like molecular properties.

### 4.10. Data Analysis

Standard bio-informatics tools and statistical parameters were implemented to guarantee the accuracy of results. All compounds were filtered through bio-availability (OB ≥ 0.3) and drug likeness (DL ≥ 0.18) thresholds. Target genes were screened for a probability of 0.1, and KEGG pathway enrichment analysis was performed with significance thresholds of *p* < 0.05. Binding affinities were recorded from the Schrodinger Glide docking score; stability and flexibility of the protein–ligand complex were analyzed through RMSD and interaction fraction, which was run for a 100 ns simulation time. Potential drug candidate toxicity and pharmacokinetic properties were evaluated using ADMET Lab 3.0 and ProTox 3.0. Also, drug-like compounds were compared with non-drug-like ones based on Lipinski’s and Pfizer’s rules.

## 5. Conclusions

Our study demonstrates that the bioactive compounds in pomegranate peels can be one of the potential therapeutic agents for pancreatic cancer. With the in silico approach, certain key compounds identified were 1-o-galloyl-beta-d-glucose, Phloridzin, epicatechin, and epicatechin gallate, which exhibit optimistic binding affinity with proteins such as AKT1, EGFR, HSP9OAA1, and PTGS2. These interactions thus indicate that bioactive compounds in pomegranate peels attack and inhibit the P13/AKT and EGFR signaling pathway, suppressing the formation of cancer cells. The significance of this study lies in the dual benefits of using pomegranate peels for the treatment of cancer with a lower risk of side effects, as well as promoting the sustainable utilization of biological waste. This study also contributes to the growing field of natural compound-based drug discovery and opens the door for new treatment strategies for pancreatic cancer, a disease with extremely poor prognosis and limited treatment options, as well as new avenues for alternative treatment strategies. Moreover, these natural compounds also demonstrated positive pharmacokinetic profiles, which complied with Lipinski criteria for drug likeness, as well as non-toxicity, for which they were evaluated as viable candidates for future drug development pipelines. By highlighting the therapeutic relevance of a widely available and underutilized natural resource, this study contributes meaningfully to the ongoing search for safer, more effective, and more accessible treatments for pancreatic cancer. However, to confirm the anticancer effects and safety profile of this compound, future research should focus on experimental validation of this compound, including studies performed both in vitro and in vivo. Further, exploring the possibility of developing these compounds into effective delivery systems and evaluating their synergistic potential with existing chemotherapies could help to further advance their development as potential candidates for clinical trials.

## Figures and Tables

**Table 1:** List of top 15 compounds and their relative information.

Compounds	CID No.	Molecular Formula	Molecular Weight (gm/mol)	Drug Likeness (DL) ≥ 0.18	Oral Bioavailabilty (OB) ≥ 0.3
Catechin	9064	C_15_H_14_O_6_	290.27	0.64	0.55
Epicatechin	72276	C_15_H_14_O_6_	290.27	0.64	0.55
Naringenin	439246	C_15_H_12_O_5_	272.25	0.82	0.55
Phloridzin	6072	C_21_H_24_O_10_	436.41	0.66	0.55
Genistein	5280961	C_15_H_10_O_5_	270.24	0.44	0.55
Gamma-Tocopherol	92729	C_28_H_48_O_2_	416.68	0.48	0.55
Daidzein	5281708	C_15_H_10_O_4_	254.24	0.29	0.55
Quinic acid	6508	C_7_H_12_O_6_	192.17	0.19	0.56
1-O-Galloyl-beta-D-glucose	124021	C_13_H_16_O_10_	332.26	0.81	0.55
Palmitelaidic acid	5282745	C_16_H_30_O_2_	254.41	0.87	0.56
Epicatechin gallate	107905	C_22_H_18_O_10_	442.37	0.93	0.55
Alpha-Zearalanol	2999413	C_18_H_26_O_5_	322.4	0.5	0.55
Beta-Zearalanol	65434	C_18_H_26_O_5_	322.4	0.5	0.55
Astragalin	5282102	C_21_H_22_O_11_	448.4	0.67	14.03
Kaempferol	5280863	C_15_H_10_O_6_	286.24	0.5	0.55

**Table 2:** Top 10 target proteins by gene degree.

Target Gene	UniProt ID	Protein Name	Degree	BC	CC
AKT1	P31749	RAC-alpha serine/threonine-protein kinase	158	0.067	0.678
IL6	P05231	Interleukin-6	157	0.066	0.678
TNF	P01375	Tumor Necrosis Factor	152	0.058	0.669
SRC	P12931	Proto-oncogene tyrosine-protein kinase Src	134	0.081	0.633
STAT3	P40763	Signal transducer and activator of transcription 3	130	0.032	0.633
EGFR	P00533	Epidermal growth factor receptor	129	0.037	0.630
BCL2	P10415	Apoptosis Regulator Bcl2	121	0.020	0.617
HSP90AA1	P07900	Heat shock protein HSP90-alpha	119	0.030	0.612
HIF1A	Q16665	Hypoxia inducible factor 1-alpha	117	0.026	0.608
PTGS2	P35354	Prostaglandin G/H synthase 2	114	0.040	0.612

BC = betweenness centrality, CC = closeness centrality.

**Table 3:** Docking score (kcal/mol) of all compounds.

Compounds	AKT1	EGFR	BCL2	HSP90AA1	PTGS2
Catechin	−6.66	−6.45	−5.61	−8.18	−7.28
Epicatechin	−6.53	−6.63	−5.82	−7.93	−7.06
Naringenin	−6.33	−6.91	−6.00	−7.85	−7.44
Phloridzin	−6.92	−6.44	−6.02	−7.08	−6.73
Genistein	−6.25	−8.10	−5.40	−5.61	−7.34
Daidzein	−5.73	−7.56	−5.11	−5.84	−7.34
Quinic acid	−4.92	−6.54	−5.04	−5.77	−6.47
1-O-Galloyl-beta-D-glucose	−6.37	−7.11	−4.92	−7.65	−6.35
Epicatechin gallate	−5.63	−6.39	−6.32	−7.92	−7.92
Astragalin	−4.42	−5.81	−6.09	−7.92	−9.00
Kaempferol	−6.25	−6.99	−6.67	−7.11	−6.96

**Table 4:** In silico ADME analysis of all compounds.

Compounds	Absorption		Distribution			Metabolism (CYP Inhibitor)					Excretion		Log S
	CaCO_2_	P-gp	HIA	BBB	VD_ss_	1A2	2C19	2C9	2D6	3A4	CL	T1/2	
**Catechin**	−6.04	–	—	—	1.15	—	—	—	—	—	14.9	2.14	−2.28
**Epicatechin**	−6.04	–	—	—	1.15	—	—	—	—	—	14.9	2.14	−2.28
**Phloridzin**	−6.20	-	—	—	0.68	—	—	—	—	++	3.76	2.26	−2.53
**Daidzein**	−4.69	-	—	—	0.62	+++	+++	+++	+++	++	7.85	1.17	−3.79
**Quinic acid**	−6.31	—	+	—	0.37	—	—	—	—	—	1.57	3.35	−0.10
**1-O-Galloyl-beta-D-glucose**	−6.39	–	+	—	0.37	—	—	—	—	—	3.68	2.37	−1.29
**Epicatechin gallate**	−6.51	—	—	—	0.44	—	—	++	—	+++	9.65	2.08	−3.70
**Kaempferol**	−5.97	–	—	—	0.15	+++	–	++	—	+++	5.69	1.33	−3.65

0–0.1 (—), 0.1–0.3 (–), 0.3–0.5 (-), 0.5–0.7 (+), 0.7–0 (++), 0.9–1.0 (+++). HIA-Human Intestinal Absorption, BBB-Blood Brain Barrier, VD_ss_-Volume distribution, CL-Clearance, Log S- Water solubility.

**Table 5:** Toxicity and Lipinski’s rule analysis of pomegranate compounds.

Compounds	Carcinogenicity	Immunotoxicity	Mutagenicity	Cytotoxicity	Lipinski	Pfizer	PAINS
Catechin	Inactive	Inactive	Inactive	Inactive	Yes	Yes	1
Epicatechin	Inactive	Inactive	Inactive	Inactive	Yes	Yes	1
Phloridzin	Inactive	Inactive	Inactive	Inactive	Yes	Yes	0
Daidzein	Inactive	Inactive	Inactive	Inactive	Yes	Yes	0
Quinic acid	Inactive	Inactive	Inactive	Inactive	Yes	Yes	0
1-O-Galloyl-beta-D-glucose	Inactive	Inactive	Inactive	Inactive	Yes	Yes	1
Epicatechin gallate	Inactive	Inactive	Inactive	Inactive	Yes	Yes	1
Kaempferol	Inactive	Inactive	Inactive	Inactive	Yes	Yes	0

## Data Availability

Data are contained within the article and Supplementary Materials.

## Supplementary Materials

The following supporting information can be downloaded at: https://www.mdpi.com/article/10.3390/ph18060896/s1, Table S1. List of 75 compounds. Table S2. Docking score of reference drugs. Figure S1. Protein secondary structure (right); α-helices (red); β-strands (blue) and rGyr (left) of five different complexes: (a) 1-O-Galloyl-beta-D-glucose with PTGS2 protein; (b) Epicatechin with HSP90AA1 protein; (c) Phloridzin with EGFR protein; (d) Epicatechin gallate with EGFR protein; (e) of 1-O-Galloyl-beta-D-glucose with AKT1 protein. Figure S2. MD simulation RMSD and Interaction fraction diagram of pomegranate peel compounds. (a–b) Quinic acid with EGFR; (c–d) Kaempferol with EGFR. Figure S3. Reference drug RMSD: (a) PTGS2 protein with Celecoxib drug; (b) HSP90AA1 protein with Tanespimycin drug; (c) EGFR protein with Erlotinib drug; (d) AKT1 protein with Afuresertib drug. Figure S4. Reference drug RMSF: (a) PTGS2 protein with Celecoxib drug; (b) HSP90AA1 protein with Tanespimycin drug; (c) EGFR protein with Erlotinib drug; (d) AKT1 protein with Afuresertib drug. Figure S5. Reference drug hydrogen bonding: (a) PTGS2 protein with Celecoxib drug; (b) HSP90AA1 protein with Tanespimycin drug; (c) EGFR protein with Erlotinib drug; (d) AKT1 protein with Afuresertib drug. Figure S6. Root Mean Square Fluctuation (RMSF) of protein alone (a) PTGS2 protein; (b) HSP90AA1 protein; (c) EGFR protein; (d) AKT1 protein. Figure S7. Molecular interaction of ligand-proteins after molecular dynamics simulation (MDS) for 5 ligands. (a) 1-O-Galloyl-beta-D-glucose with PTGS2 protein; (b) Epicatechin with HSP90AA1 protein; (c) Phloridzin with EGFR protein; (d) Epicatechin gallate with EGFR protein; (e) of 1-O-Galloyl-beta-D-glucose with AKT1 protein.

## Abbreviations

The following abbreviations are used in this manuscript:

PC	Pancreatic cancer
PG	Pomegranate
PP	Pomegranate peel
GO	Gene ontology
KEGG	Kyoto Encyclopedia of genes and genomes
DL	Drug likeness
BO	Bio-availability
PPI	Protein–protein interaction
STRING	Search tool for the retrieval of interacting genes/proteins
DC	Degree of connectivity
CC	Closeness centrality
BP	Biological processes
MF	Molecular function
DAVID	Database for annotation, visualization, and integrated discovery
RCSB	Research collaboratory for structural bioinformatics
PDB	Protein data bank
RMSD	Root mean square deviation
RMSF	Root mean square fluctuation
AKT1	RAC-alpha serine/threonine-protein kinase
IL6	Interleukin-6
TNF	Tumor necrosis factor
STAT3	Signal transducer and activator of transcription 3
EGFR	Epidermal growth factor receptor
BCL2	Apoptosis regulator Bcl2
HSP90AA1	Heat shock protein HSP90-alpha
HIF1A	Hypoxia-inducible factor 1-alpha
PTGS2	Prostaglandin G/H synthase 2
