# Supplementary material for: Harnessing the Therapeutic Potential of Pomegranate Peel-Derived Bioactive Compounds in Pancreatic Cancer: A Computational Approach
Source: Pharmaceuticals (Basel). 2025 Jun 15;18(6):896. doi: 10.3390/ph18060896 (PMC12195666; doi:10.3390/ph18060896)
Supplement: Supplementary file 1 [file pharmaceuticals-18-00896-s001.zip › pharmaceuticals-3668070-supplementary.pdf]

Harnessing the therapeutic potential of pomegranate peel derived bioactive compounds in pancreatic cancer: A computational approach

Rita Majhi[1,+], Sagar Kurmi[1,+], Hilal Tayara[2,*] Kil To Chong[1,3,*]

[1]Department of Electronics and Information Engineering, Jeonbuk National University, Jeonju-si, 54896, Jeollabuk-do, Korea

[2]School of International Engineering and Science, Jeonbuk National University, Jeonju-si, 54896, Jeollabuk-do, Korea

[3]Advanced Electronics and Information Research Center, Jeonbuk National University, Jeonju-si, 54896, Jeollabuk-do, Korea

*** Correspondence:** kitchong@jbnu.ac.kr (K.T.C.); hilaltayara@jbnu.ac.kr (H.T.)

+ These authors contributed equally to this work.

Table S1. List of 75 compounds.

Compounds	Molecular Formula	Compounds	Molecular Formula
Betulinic acid	$C_{30}H_{48}O_3$	Isolariciresinol	$C_{20}H_{24}O_6$
Asiatic acid	$C_{30}H_{48}O_5$	alpha-Conidendrin	$C_{20}H_{20}O_6$
Ursolic acid	$C_{30}H_{48}O_3$	(+)-Dihydrokaempferol	$C_{15}H_{12}O_6$
Maslinic acid	$C_{30}H_{48}O_4$	Leucocyanidin	$C_{15}H_{14}O_7$
beta-Sitosterol	$C_{29}H_{50}O$	gamma-Tocopherol	$C_{28}H_{48}O_2$
Citric acid	$C_6H_8O_7$	Daidzein	$C_{15}H_{10}O_4$
Hexanal	$C_6H_{12}O$	Oleanolic acid	$C_{30}H_{48}O_3$
Ascorbic acid	$C_6H_8O_6$	Leucopelargonidin	$C_{15}H_{14}O_6$
beta-Ionone	$C_{13}H_{20}O$	Acetylursolic acid	$C_{32}H_{50}O_4$
Nortrachelogenin	$C_{20}H_{22}O_7$	2alpha,3beta-Dihydroxyolean-12-en-28-oic acid	$C_{30}H_{48}O_4$
(+)-Lariciresinol	$C_{20}H_{24}O_6$	Punicanolic acid	$C_{30}H_{50}O_4$
5,7,3',4'-Tetrahydroxyflavone	$C_{15}H_{10}O_6$	Ursonic acid	$C_{30}H_{46}O_3$
Dihydroquercetin	$C_{15}H_{12}O_7$	Quinic acid	$C_7H_{12}O_6$
Ampelopsin	$C_{15}H_{12}O_8$	1-O-Galloyl-beta-D-glucose	$C_{13}H_{16}O_{10}$
(+)-Catechin	$C_{15}H_{14}O_6$	7-Hydroxymatairesinol	$C_{20}H_{22}O_7$
(-)-Epicatechin	$C_{15}H_{14}O_6$	Isohydroxymatairesinol	$C_{20}H_{22}O_7$
Eriodictyol	$C_{15}H_{12}O_6$	Pregnenolone	$C_{21}H_{32}O_2$
Hesperetin	$C_{16}H_{14}O_6$	Palmitelaidic acid	$C_{16}H_{30}O_2$
Naringenin	$C_{15}H_{12}O_5$	Epicatechin gallate	$C_{22}H_{18}O_{10}$
Phloridzin	$C_{21}H_{24}O_{10}$	alpha-Zearalanol	$C_{18}H_{26}O_5$
Dihydrochrysin	$C_{15}H_{12}O_4$	Estradiol	$C_{18}H_{24}O_2$
Apigenin 7-O-beta-D-glucopyranoside	$C_{21}H_{20}O_{10}$	Ursolic acid methyl ester	$C_{31}H_{50}O_3$
Caffeine	$C_8H_{10}N_4O_2$	alpha-Zearalenol	$C_{18}H_{24}O_5$
Genistein	$C_{15}H_{10}O_5$	beta-Zearalanol	$C_{18}H_{26}O_5$
Glucogallin	$C_{13}H_{16}O_{10}$	p-Menth-1-en-3-one semicarbazone	$C_{11}H_{19}N_{3O}$
Labiatenic acid	$C_{18}H_{16}O_8$	Estriol	$C_{18}H_{24}O_3$
Campesterol	$C_{28}H_{48}O$	Dihydroflavonol	$C_{15}H_{12}O_3$
Cholesterol	$C_{27}H_{46}O$	alpha-Estradiol	$C_{18}H_{24}O_2$
Oestrone	$C_{18}H_{22}O_2$	beta-Zearalenol	$C_{18}H_{24}O_5$
(-)-beta-Sitosterol	$C_{29}H_{50}O$	PELTATIN B	$C_{22}H_{22}O_8$
beta-Stigmasterol	$C_{29}H_{48}O$	Hirsutrin	$C_{21}H_{20}O_{12}$
beta-Betulinic acid	$C_{30}H_{48}O_3$	Astragalin	$C_{21}H_{20}O_{11}$
Kaempferol	$C_{15}H_{10}O_6$	luteolin	$C_{15}H_{10}O_6$
Quercetin	$C_{15}H_{10}O_7$	officinalisin	$C_{50}H_{84}O_{23}$
Afzelin	$C_{21}H_{20}O_{10}$		

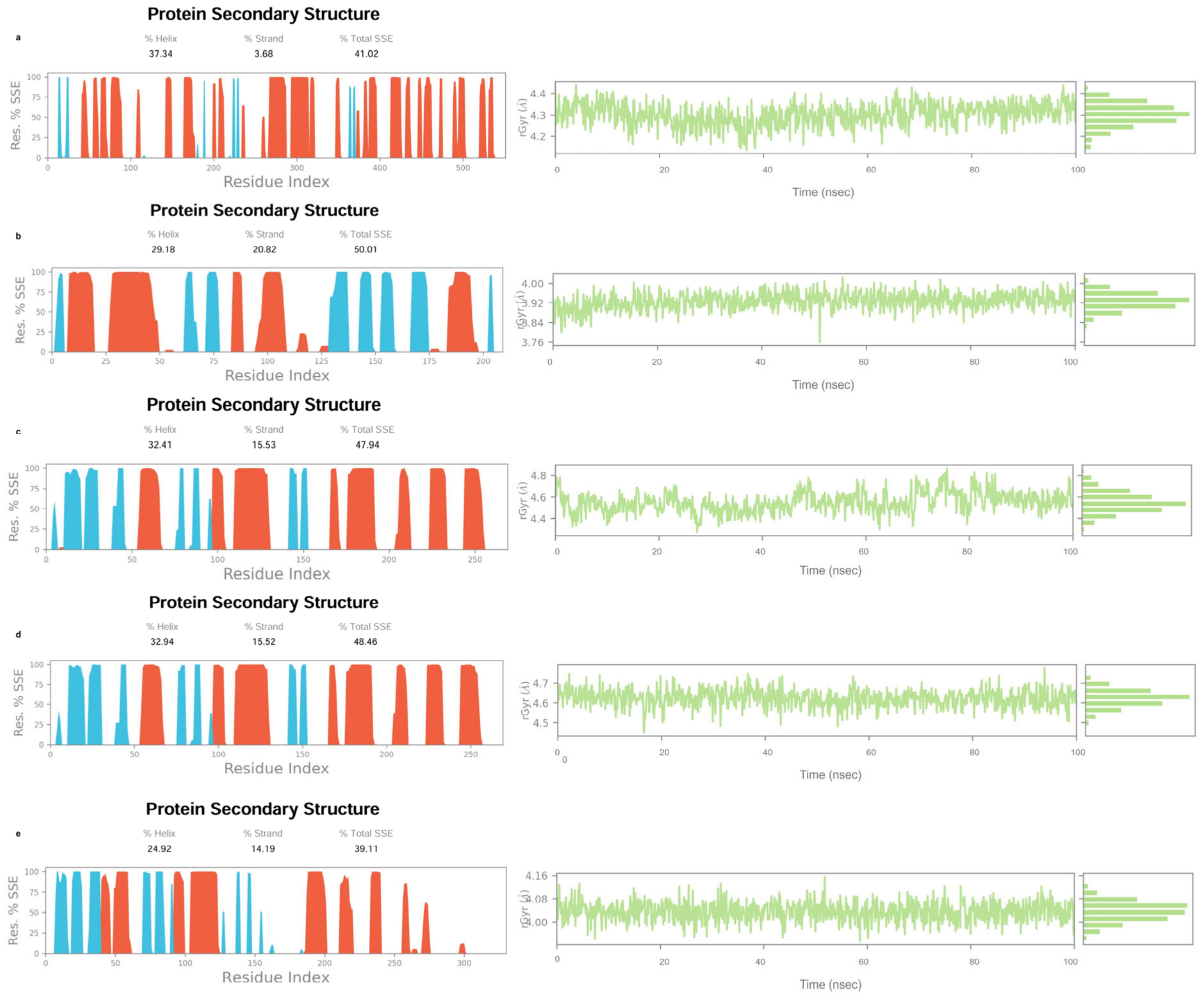

Figure S1. Protein secondary structure (right); α-helices (red); β-strands (blue) and rGyr (left) of five different complexes: (a) 1-O-Galloyl-beta-D-glucose with PTGS2 protein; (b) Epicatechin with HSP90AA1 protein; (c) Phloridzin with EGFR protein; (d) Epicatechin gallate with EGFR protein; (e) of 1-O-Galloyl-beta-D-glucose with AKT1 protein.

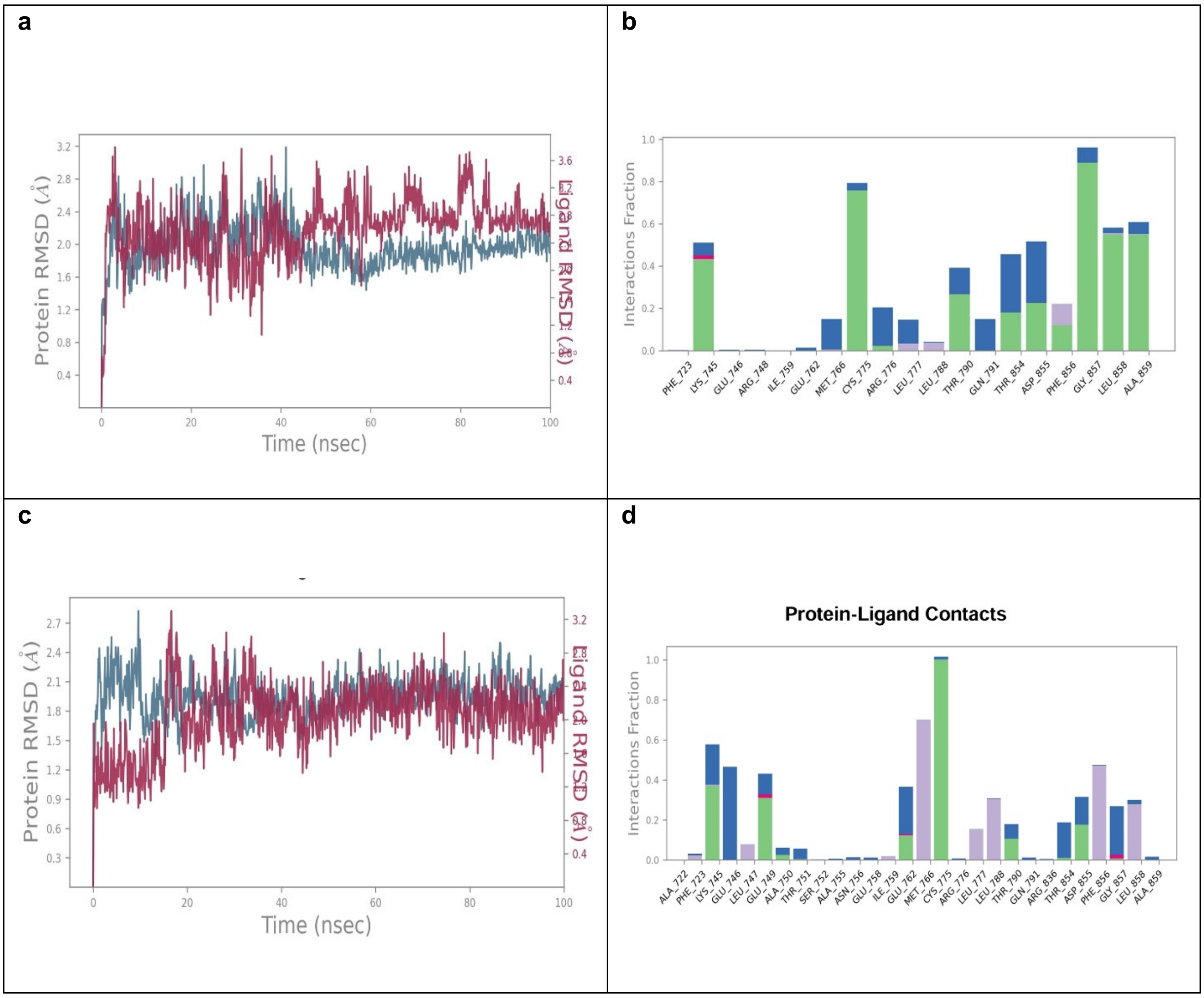

Figure S2. MD simulation RMSD and Interaction fraction diagram of pomegranate peel compounds. (a-b) Quinic acid with EGFR; (c-d) Kaempferol with EGFR.

Table S2. Docking score of reference drugs.

**Proteins**	**Reference drugs**	**Docking score (kcal/mol)**
PTGS2	Celecoxib	-7.8
HSP90AA1	Tanespimycin	-8.9
EGFR	Erlotinib	-8.9
AKT1	Afuresertib	-8.1

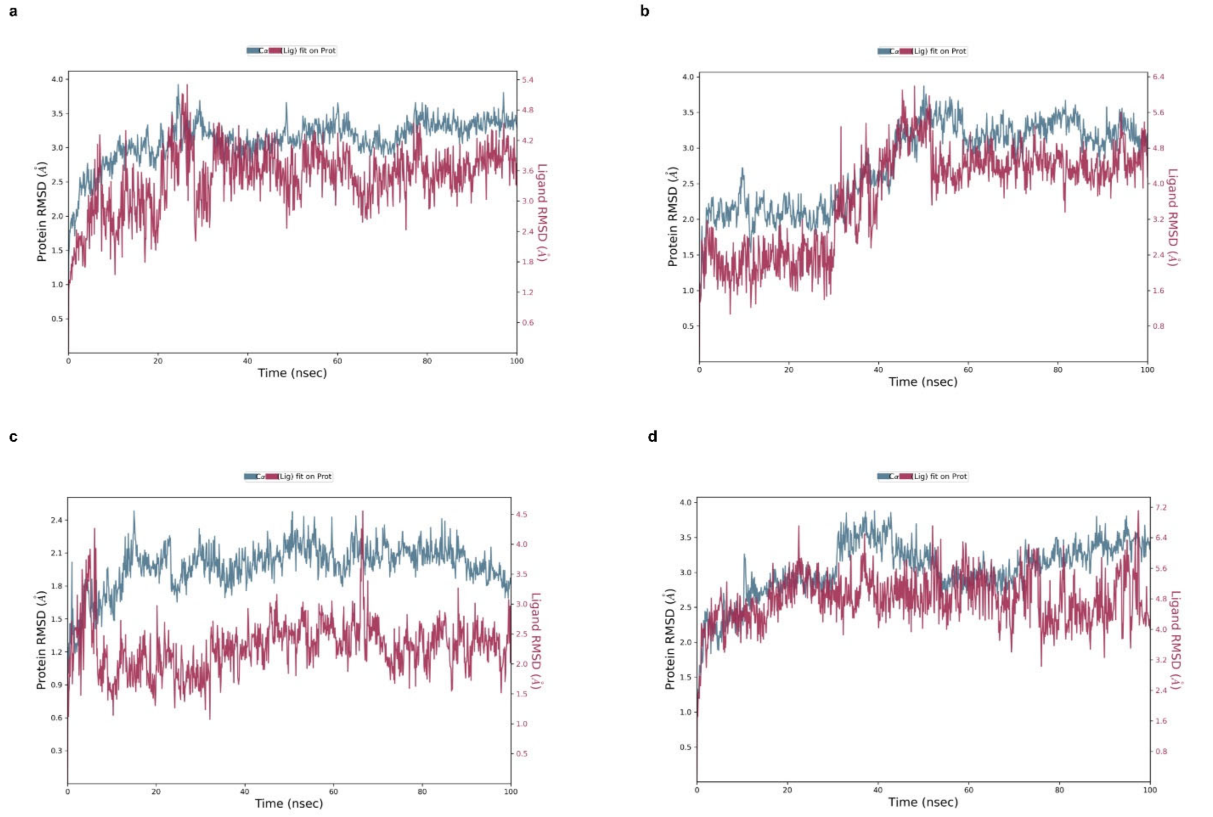

Figure S3. Reference drug RMSD :(a) PTGS2 protein with Celecoxib drug; (b) HSP90AA1 protein with Tanespimycin drug; (c) EGFR protein with Erlotinib drug; (d) AKT1 protein with Afuresertib drug.

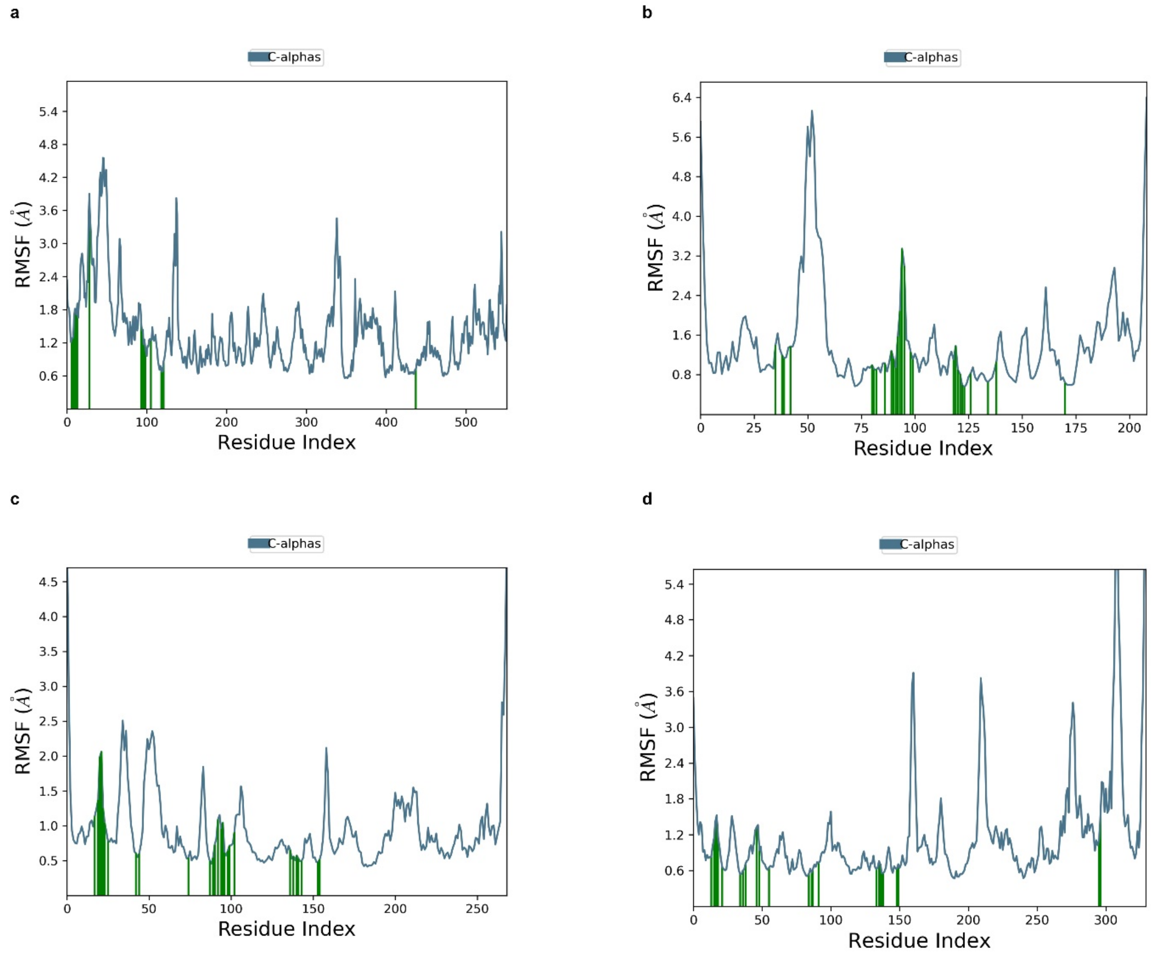

Figure S4. Reference drug RMSF: (a) PTGS2 protein with Celecoxib drug; (b) HSP90AA1 protein with Tanespimycin drug; (c) EGFR protein with Erlotinib drug; (d) AKT1 protein with Afuresertib drug.

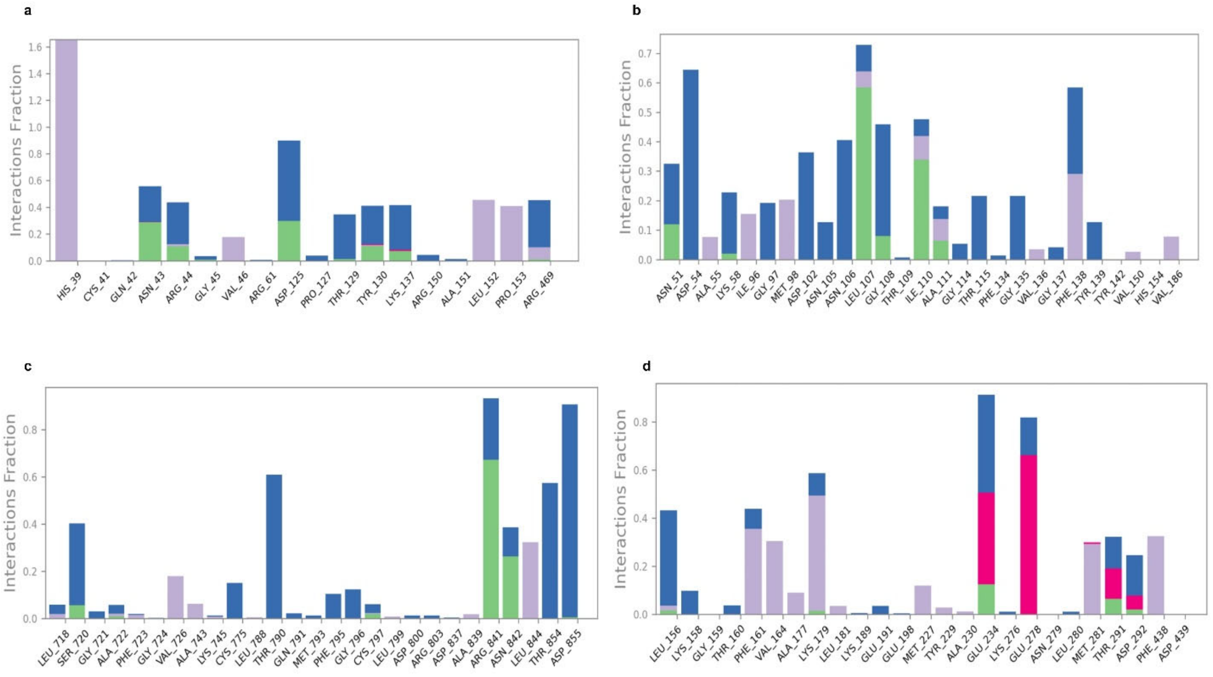

Figure S5. Reference drug hydrogen bonding : (a) PTGS2 protein with Celecoxib drug; (b) HSP90AA1 protein with Tanespimycin drug; (c) EGFR protein with Erlotinib drug; (d) AKT1 protein with Afuresertib drug.

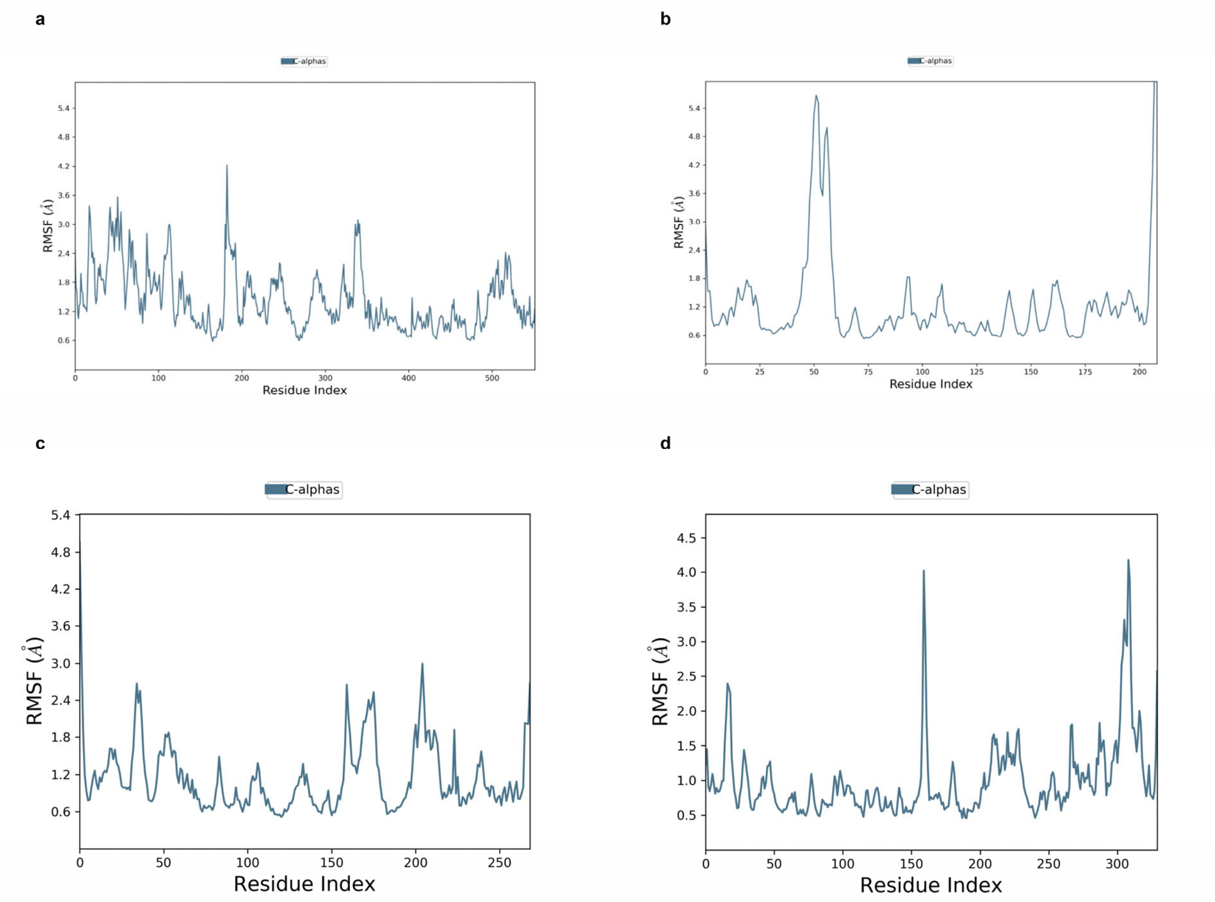

Figure S6. Root Mean Square Fluctuation (RMSF) of protein alone (a) PTGS2 protein; (b) HSP90AA1 protein; (c) EGFR protein; (d) AKT1 protein.

Description of Figure S6:

The apo protein RMSF has higher flexibility (Figure S6 (a)) over 100 ns simulation particularly in the N-terminal and loop regions whereas the complex (1-O-Galloyl-beta-D-glucose with PTGS2 protein) exhibited lesser fluctuations in key regions, particularly at residues 180–220 and 350– 420 indicating that ligand binding enhances protein structure stabilization. (Figure S6 (b)), the higher instability was observed between residues ~35–65 of apo protein, might causes enhancement in flexibility at these regions. Resulting inherent dynamic nature of the unbound protein. On the other hand, epicatechin with HSP90AA1 protein complex (Figure 7 (b)) displayed reduced fluctuations across the same regions and at residues 40–60 and 190- 200, highlighting movement restriction of key loops and active-site-adjacent.

The Figure S7 (c) showed intrinsic flexibility in regions such as residues 35–45 (~2.6 Å) and 200–215 (~3.0 Å) and upon ligand contact (Figure 7 (c)), we observed notable changes in fluctuation patterns. For instance, residues 145–155 exhibit increased flexibility (~3.6 Å), suggesting a ligand-induced dynamic effect, while residues 35–45 and 200–215 show reduced mobility, indicating stabilization, emphasize that ligand interaction leads to both localized flexibility and rigidity. Similarly, in Figure 7 (d) several regions showed significant shift in fluctuation in ligand bound protein. There was marked increase in RMSF (~3.2 Å) at 140–150 residues which is due to ligand effect on flexibility or partial destabilization. In contrast, some reason become more stable (35–45 and 2050) with reduced fluctuations in the holo state. Figure S6 (d), ligand-bound structure displayed elevated variations at residues 285–295 and 310–320 reached peaks above 4.5 Å, indicating enhanced mobility in these distal regions potentially due to allosteric effects while apo protein fluctuations generally remained below 1.5 Å but some residues showed slightly fluctuation to 2.5-3.0 Å. These results demonstrate that ligand binding not only stabilizes specific binding-site residues but also induces long-range dynamic changes across the protein structure, highlighting the allosteric nature of the interaction.

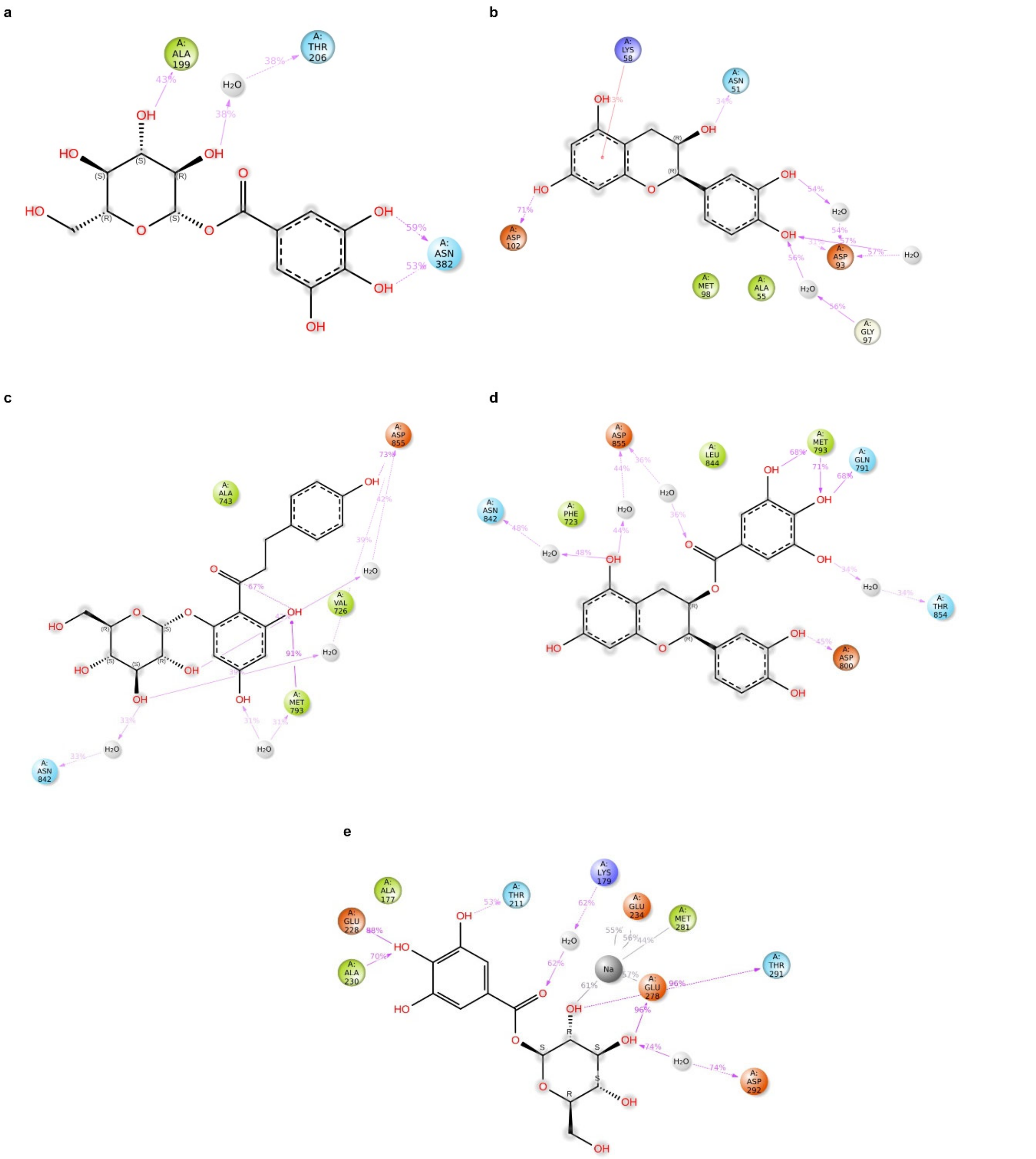

Figure S7. Molecular interaction of ligand-proteins after molecular dynamics simulation (MDS) for 5 ligands. (a) 1-O-Galloyl-beta-D-glucose with PTGS2 protein; (b) Epicatechin with HSP90AA1 protein; (c) Phloridzin with EGFR protein; (d) Epicatechin gallate with EGFR protein; (e) of 1-O-Galloyl-beta-D-glucose with AKT1 protein.
